# Identification of a gene encoding polygalacturonase expressed specifically in short styles in distylous common buckwheat (*Fagopyrum esculentum*)

**DOI:** 10.1038/s41437-019-0227-x

**Published:** 2019-05-10

**Authors:** Ryoma Takeshima, Takeshi Nishio, Setsuko Komatsu, Nobuyuki Kurauchi, Katsuhiro Matsui

**Affiliations:** 1 Institute of Crop Science, National Agriculture and Food Research Organization (NARO), Kannondai 2-1-2, Tsukuba, Ibaraki 305-8518 Japan; 2 Tohoku University, Aoba-ku, Sendai 980-0845 Japan; 3 Department of Environmental and Food Sciences, Fukui University of Technology, Gakuen 3-6-1, Fukui, 910-8505 Japan; 4 College of Bioresource Sciences, Nihon University, 1866, Kameino, Fujisawa, Kanagawa 252-0880 Japan; 5 Graduate School of Life and Environmental Science, University of Tsukuba, Kannondai 2-1-2, Tsukuba, Ibaraki 305-8518 Japan

**Keywords:** Genetic linkage study, Plant molecular biology

## Abstract

Common buckwheat (*Fagopyrum esculentum*) is a heteromorphic self-incompatible (SI) species with two types of floral architecture: thrum (short style) and pin (long style). The floral morphology and intra-morph incompatibility are controlled by a single genetic locus, *S*. However, the molecular mechanisms underlying the heteromorphic self-incompatibility of common buckwheat remain unclear. To identify these mechanisms, we performed proteomic, quantitative reverse-transcription PCR, and linkage analyses. Comparison of protein profiles between the long and short styles revealed a protein unique to the short style. Amino-acid sequencing revealed that it was a truncated form of polygalacturonase (PG); we designated the gene encoding this protein *FePG1*. Phylogenetic analysis classified FePG1 into the same clade as PGs that function in pollen development and floral morphology. *FePG1* expression was significantly higher in short styles than in long styles. It was expressed in flowers of a short-homostyle line but not in flowers of a long-homostyle line. Linkage analysis indicated that *FePG1* was not linked to the *S* locus; it could be a factor downstream of this locus. Our finding of a gene putatively working under the regulation of the *S* locus provides useful information for elucidation of the mechanism of heteromorphic self-incompatibility.

## Introduction

In many flowering plants, self-incompatibility is important in preventing inbreeding and promoting outbreeding. Self-incompatibility is classified according to whether it is associated with morphological differences in style length and anther height (heteromorphic self-incompatibility) or not (homomorphic self-incompatibility) (De Nettancourt 1997; Barrett 2002).

**Fig. 1:**
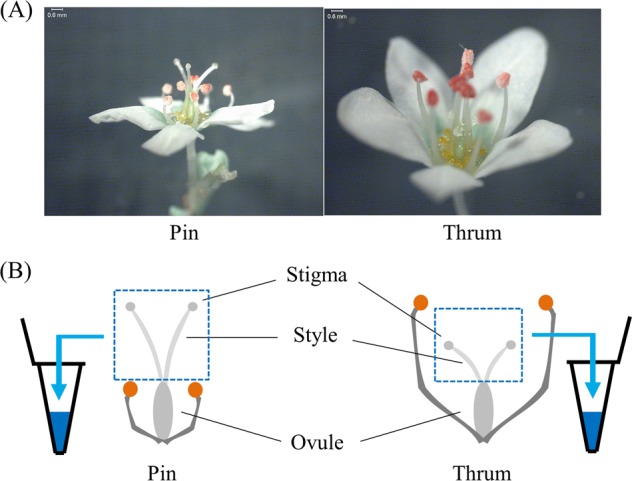
Floral morphotypes of common buckwheat (*Fagopyrum esculentum*) and method of sampling for 2D-PAGE analysis. **a** Pin flower has long styles and low anthers (left panel); thrum flower has short styles and high anthers (right panel). **b** Sampling for 2D-PAGE. The stigmas and styles separated from ovules were collected

Common buckwheat (*Fagopyrum esculentum*) is a heteromorphic self-incompatible (SI) species with two types of floral architecture: thrum (short style, high anthers, and large pollen grains) and pin (long style, low anthers, and small pollen grains; Fig. 1a). This characteristic is controlled by alleles at a single genetic locus, *S*, which segregates as a simple Mendelian factor, thrum being heterozygous *Ss* and pin being homozygous recessive *ss* (*e.g*., Garber and Quisenberry 1927; Lewis and Jones 1992). Sharma and Boyes (1961) considered that the *S* locus of common buckwheat is similar to the *S* locus proposed to occur in distylous *Primula* (Dowrick 1956). They postulated that the *S* locus (*S* supergene) of common buckwheat consists of five genes: *G*, style length; *I*^*S*^, stylar incompatibility; *I*^*P*^, pollen incompatibility; *P*, pollen size; and *A*, anther height. The genotype of pin (*ss*) is considered to be *g i*^*s*^*i*^*p*^ *pa*/*g i*^*s*^*i*^*p*^ *pa* and that of thrum (*Ss*) is considered to be *G I*^*S*^*I*^*P*^ *PA*/*g i*^*s*^*i*^*p*^ *pa*; however, these five genes have not yet been identified (Matsui et al. 2007).

Some buckwheat species are self-compatible (SC), such as *Fagopyrum homotropicum*, as discovered by Ohnishi (1998). An interspecific cross between *F. esculentum* and *F. homotropicum* enabled the production of SC lines, which can be crossed with common buckwheat lines (Campbell 1995; Aii et al. 1998; Woo et al. 1999; Matsui et al. 2003). The SC allele is designated *S*^*h*^; the dominance relationship is *S* > *S*^*h*^ > *s* (Woo et al. 1999).

By comparing bacterial artificial chromosome contigs spanning the *S* locus and partial genome sequence contigs obtained from different flower types, Li et al. (2016) assembled the *S* locus of *Primula vulgaris* and clarified that the *S* supergene consists of tightly linked five predicted genes and is present only in the genome of thrum plants. By comparing worldwide landraces of common buckwheat and two distantly related heteromorphic SI species, Yasui et al. (2012) found that a genomic region around the *S-LOCUS EARLY FLOWERING 3* (*S*-*ELF3*) gene is absent in the *s* haplotype of common buckwheat, and that the *S*-*ELF3* gene is tightly linked to the *S* locus; however, the functions of *S-ELF3* and other genes at the *S* locus remain unclear.

In the *Brassicaceae*, *Solanaceae*, and *Papaveraceae*, identification of proteins segregating with *S* haplotypes has led to the isolation of the *S* genes (reviewed by Takayama and Isogai 2005; Franklin-Tong 2008). Protein-level analyses have also been carried out in *Primula* (Golynskaya et al. 1976; Shivanna et al. 1981), *Averrhoa carambola* (Wong et al. 1994), *Turnera subulata* (Athanasiou et al. 2003; Tamari and Shore 2006), common buckwheat (Miljus-Dukic et al. 2004), and *Linum grandiflorum* (Ushijima et al. 2012), and genes related to heteromorphic self-incompatibility have been identified in *T. subulata* and *L. grandiflorum*. In *T. subulata*, Athanasiou and Shore (1997) detected thrum-specific style and pollen proteins by conducting isoelectric focusing of non-denatured proteins. Athanasiou et al. (2003) subsequently identified the amino acid sequences of thrum-specific proteins. One protein was a member of the polygalacturonase (PG) family, which comprises pectin hydrolytic enzymes involved in development of pollen, anther, and floral morphology (Zhang et al. 2008; Huang et al. 2009a, b); cell elongation (Babu et al. 2013); seed germination (Sitrit et al. 1996, 1999); pod and anther dehiscence (Ogawa et al. 2009; Xiao et al. 2014); and fruit softening (Kramer and Redenbaugh 1994). Ushijima et al. (2012) detected four predicted heterostyly-related genes in *L. grandiflorum*: *thrum style-specific gene 1* (*TSS1*), *LgAP1*, *LgMYB21*, and *LgGLX1*. They suggested *TSS1* as a candidate gene within the *S* supergene, but this is unresolved. In common buckwheat, Miljus-Dukic et al. (2004) detected some protein spots that specifically appeared in either pin or thrum by 2D-PAGE, but no proteins were identified.

Here we performed proteomic, expression, and genetic linkage analyses to discover genes related to the heteromorphic self-incompatibility in common buckwheat. We detected a specific protein in short styles and identified its gene as a PG homologue; we designated it *FePG1*. Our results indicate that *FePG1* is not a gene within the *S* supergene but it could act downstream of the *S* locus.

## Materials and methods

### Plant materials

Plant materials are listed in Supplementary Table S1. For the proteomic analysis, we used four Japanese cultivars, ‘Botansoba’ (BTN), ‘Hashikamiwase’, ‘Hitachiakisoba’ (HTC), and ‘Shinano 1’; three Japanese landraces, ‘Asahimura-zairai 3’, ‘Kanoya-zairai’, and ‘Kugino-zairai’; and a SC long-homostyle line, ‘Norin-PL1’ (Matsui et al. 2008). For the semi-quantitative reverse transcription PCR (semi-qRT-PCR) analysis, we used the Japanese cultivar ‘Sachiizumi’, the SC long-homostyle line ‘Kyukei SC7’ (KSC7) developed from ‘Norin-PL1’, and the short-homostyle line ‘L21SH’ (F_7_ of ‘BTN’ × ‘Pennline 10’; Marshall 1970). For the real-time qRT-PCR analysis, we used two Japanese cultivars, ‘Kitawasesoba’ and ‘HTC’, and a Chinese cultivar, ‘CM 221’. ‘Kitawasesoba’ and ‘CM 221’ are both summer ecotypes, whereas ‘HTC’ is a medium-autumn ecotype. Styles were sampled in summer. For linkage analysis, we used two F_2_ populations derived from a ‘Kyukei 28’ × ‘KSC7’ cross (*n* = 130) and a ‘Kyushu 7’ × ‘KSC7’ cross (*n* = 97).

### Two-dimensional polyacrylamide gel electrophoresis

Each pistil was separated into the upper part (including the stigmas and styles) and the lower part (including the ovule). For 2D-PAGE, the upper parts of 20 pistils (Fig. 1b) were combined and homogenised in lysis buffer (O’Farrell 1975) containing 8 M urea, 2% Nonidet P-40, 0.8% Ampholine (pH 3.5–10), 0.8% Ampholine (pH 5–8), 5% 2-mercaptoethanol, and 5% polyvinyl pyrrolidone, and centrifuged twice at 10,000 × *g* for 5 min. The supernatant was used as samples for 2D-PAGE with isoelectric focusing in the first dimension and SDS-PAGE in the second dimension (O’Farrell 1975). Searches for short-style–specific proteins were conducted in the pH range of 4–7. The gels were silver-stained with a Sil-Best staining kit (Nacalai Tesque, Kyoto, Japan).

### N-terminal and internal amino acid sequencing

The upper parts of 100 pistils per floral morphotype were homogenised in lysis buffer, separated by 2D-PAGE and stained with Coomassie Brilliant Blue. Five protein spots were excised with a razor, eluted and separated by SDS-PAGE. The proteins were electro-blotted onto a polyvinylidene difluoride membrane and detected by Coomassie Brilliant Blue staining. Stained proteins were excised from the membrane and directly subjected to Edman degradation on a gas-phase protein sequencer (Procise clC 492, Applied Biosystems, Foster City, CA, USA).

To determine the amino acid sequences of internal peptides, the proteins in the excised gel pieces were digested with trypsin and the digests were separated by reverse-phase HPLC. HPLC analysis was carried out using a Symmetry C18 column (3.5 μm, 1.0 × 150 mm; Waters) at a flow rate of 50 μL/min. The mobile phase comprised two kinds of solvent (A, 2% acetonitrile containing 0.1% TFA; B, 90% acetonitrile containing 0.09% TFA). We used the following gradient: from 100% A plus 6% B at 0 min to 100% B plus at 91 min. The two digested fractions were sequenced on a Procise clC 494 protein sequencer.

### Cloning a gene encoding the short-style–specific protein

Because the short style is controlled by the *S* allele (see the Introduction), thrum plants of ‘HTC’ were used for cloning. Genomic DNA was isolated from young leaves with a DNeasy Plant Mini Kit (Qiagen, Hilden, Germany). Total RNA was isolated from buds, mRNA was isolated from total RNA with a Micro-Fast Track Kit (Invitrogen, Carlsbad, CA, USA), and cDNA was synthesised with the Universal Ribo Clone cDNA synthesis system (Promega, Madison, WI, USA). Based on the N-terminal and internal partial amino acid sequences, degenerate primers were designed and used to obtain partial cDNA and genomic DNA fragments. Thermocycling conditions were as follows: initial denaturation at 94 °C for 2 min; 35 cycles of 94 °C for 30 s, 42 °C for 30 s, 72 °C for 1 min; and final extension at 72 °C for 5 min. Amplified DNA fragments were cloned with the TA Cloning Kit (Invitrogen) and sequenced on an ABI3100 sequencer (Applied Biosystems, Waltham, MA, USA). We performed 3ʹ RACE with the 3ʹ-Full RACE Core Set (Takara, Otsu, Japan) to determine the sequence of the 3ʹ region of the gene, and then conducted genome walking with the GeneRacer Kit (Invitrogen) to determine the sequence of the 5ʹ region of the gene. Primers are listed in Supplementary Table S2.

### Tandem mass spectrometry and *de novo* sequence analysis

To confirm that the DNA sequence identified by the 3ʹ RACE and genome walking encodes the short-style–specific protein, we obtained internal partial amino acid sequences de novo by tandem mass spectrometry (MS/MS) with ESI-Q-TOF (Waters, Milford, USA).

### Bioinformatics analysis

We searched the National Center for Biotechnology Information (NCBI) database (https://www.ncbi.nlm.nih.gov/) and the Buckwheat Genome Data Base (BGDB; http://buckwheat.kazusa.or.jp; Yasui et al. 2016). The estimated molecular weight and isoelectric point (pI) of the thrum-specific protein were calculated by ExPASy (https://www.expasy.org/). Deduced amino acid sequences of 58 PG orthologues from various plant species were retrieved from BGDB and GenBank (Supplementary Tables S3 and S4). Multiple alignment of the glycoside hydrolase family 28 domain sequences of the 58 PGs was performed using the MAFFT version 7 online service (Katoh and Standley 2013; https://mafft.cbrc.jp/alignment/server/). A phylogenetic tree was generated in MEGA X software (Kumar et al. 2018) using the maximum likelihood method and Jones–Taylor–Thornton matrix–based model with 1000 bootstrap repetitions. Promoter search was performed in PlantPAN 3.0 software (Chow et al. 2019; http://plantpan.itps.ncku.edu.tw/).

### RNA isolation and qRT-PCR

Total RNA was isolated from leaves, stems, roots, whole open flowers, and styles with the RNeasy Plant Mini Kit (Qiagen). Genomic DNA was removed using DNase I (Thermo Fisher Scientific, Waltham, MA, USA). cDNA was synthesised from 600 ng DNase I-treated total RNA with the iScript cDNA Synthesis Kit (Bio-Rad, Hercules, CA, USA). Semi-qRT-PCR was performed using *Ex-Taq* DNA polymerase (Takara). Thermocycling conditions were 94 °C for 2 min; 40 cycles of 94 °C for 30 s, 58 °C for 30 s, 72 °C for 30 s; and a final 72 °C for 5 min. Real-time qRT-PCR was performed using iQ Green Supermix (Bio-Rad). Thermocycling conditions were 95 °C for 3 min, followed by 40 cycles of 95 °C for 10 s, 58.5 °C for 20 s, and 72 °C for 30 s. *Histone H3* (*H3*) was used as an internal control. For each transcript, amplification of a single DNA fragment was confirmed by melt curve analysis and agarose gel electrophoresis of the PCR products. Primers are listed in Supplementary Table S2.

### Linkage analysis

Genomic DNA was isolated from young leaves with a DNeasy Plant Mini Kit. To clarify whether the *FePG1* locus is linked to the *S* locus, linkage analysis was performed using two F_2_ populations derived from a ‘Kyukei 28’ × ‘KSC7’ cross (*n* = 130) and a ‘Kyushu 7’ × ‘KSC7’ cross (*n* = 97). A DNA marker to distinguish the genotype was designed at the position of the 692-bp insertion in the 3rd exon of *FePG1* (Supplementary Table S2). The amplified products were separated in 1% agarose gel. Segregations of floral morphotypes (pin or long homostyle) and genotypes of *FePG1* were analysed.

## Results

### Identification of short-style–specific proteins

**Fig. 2:**
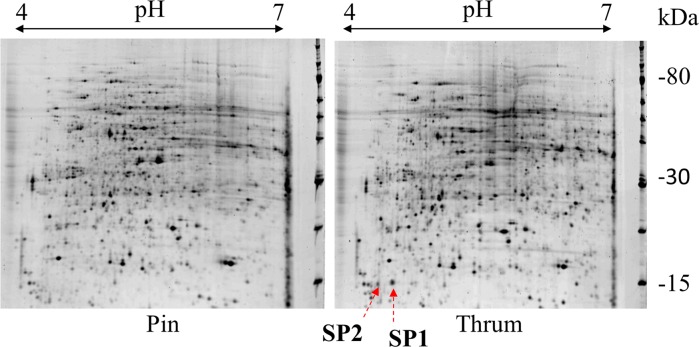
2D-PAGE analysis of proteins extracted from upper parts of pistils of pin and thrum plants. Total proteins from stigmas and styles were separated by 2D-PAGE and silver-stained. SP1 and SP2, thrum-specific spots

By comparing protein profiles of the upper pistils of pin and thrum plants by 2D-PAGE analysis (Fig. 2), we detected two thrum-specific (short-style–specific) spots corresponding to ca. 15-kDa proteins. Spot 1 (SP1) accumulated highly and specifically in the thrum upper pistils in all four cultivars and three Japanese landraces tested, whereas Spot 2 (SP2) accumulated at a low level and was sometimes absent in the thrum upper pistils. No SP1 or SP2 was detected in the styles of the long-homostyle line ‘Norin-PL1’.

**Fig. 3:**
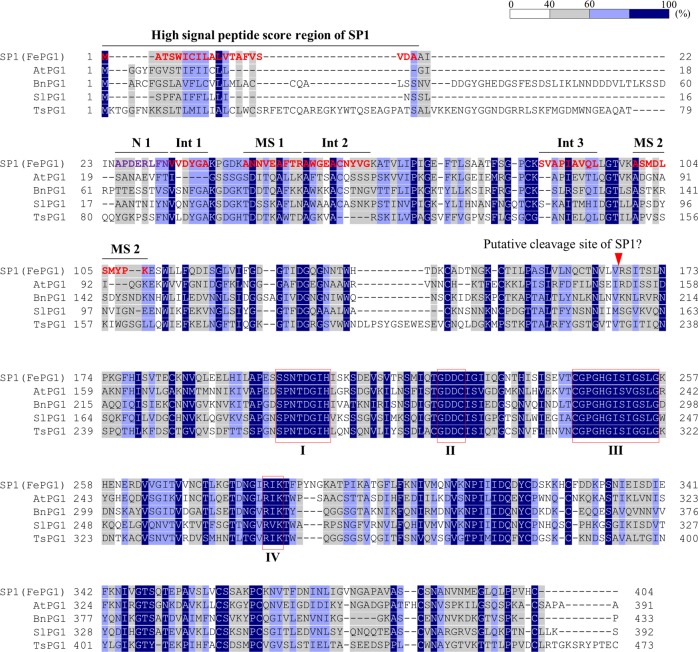
Amino-acid sequence of SP1 (FePG1) and multiple sequence alignment analysis. The predicted amino-acid sequence of FePG1 was aligned with those of four PG orthologues from *Arabidopsis thaliana* (At), *Brassica napus* (Bn), *Solanum lycopersicum* (Sl), and *Turnera subulata* (Ts). Conserved domains of PGs are indicated as regions I–IV. N-terminal and internal amino-acid sequences obtained by Edman sequencing and MS/MS analysis are indicated as N1, Int1, Int2, MS1, and MS2. The putative cleavage site was predicted by ExPASy

We obtained the same N-terminal amino acid sequence (APDERLFNV) for SP1 and SP2 (Fig. 3). Therefore, a post-translational modification, such as phosphorylation, might have caused the difference in pI points between SP1 and SP2. Because of the stability of SP1 in the styles, all further experiments were performed using SP1.

To determine internal amino acid sequences, the SP1 protein was digested with trypsin, and three different amino acid sequences (LFNVVDYGA, SIAPIAVQL, and AWGEACNYVG) were obtained. According to these four sequences, we developed degenerate primers (Supplementary Table S2) and identified a partial ca. 100-bp nucleotide sequence of the gene encoding SP1 (Supplementary Table S2). The full-length sequence determined by 3ʹ RACE and genome walking showed that *SP1* has a 1,215-bp coding sequence that encodes 404 amino acids (Fig. 3).

To confirm that this deduced sequence corresponds to SP1, we performed MS/MS analysis of SP1 and obtained two amino-acid sequences, ANNVEAFTR and ASMDLSMYPK. Both these sequences were in the deduced amino acid sequence of SP1 (Fig. 3).

In 2D-PAGE analysis, SP1 was detected at the position of 15 kDa and pI 4.7 (Fig. 2). However, the estimated molecular mass and pI of the putative SP1 protein were 43 kDa and 5.38, respectively, indicating that SP1 is post-translationally modified. By comparing the N-terminal sequence and deduced full-length amino acid sequence, we found that SP1 had a signal peptide, MATSWICILALVTAFVSVDAAIIN (Fig. 3). We calculated the molecular mass and pI of putative mature proteins spanning from the N-terminal sequence (Fig. 3; APDERLFNV) to predicted C-terminal points in ExPASy (https://www.expasy.org/). To the 166th valine, they were 15.1 kDa and 4.78; to the 167th arginine they were 15.26 kDa and 4.99. From the molecular mass and pI of SP1 estimated by 2D-PAGE analysis, we suggest that SP1 may be cleaved between the 166th and 167th residues (Fig. 3). However, because estimation of molecular mass and pI by 2D-PAGE is not very accurate, further analysis is required to confirm the cleavage position.

### Database search and phylogenetic analysis of SP1

BLASTP search of the NCBI database showed that the predicted full-length SP1 was similar to PG-like proteins of other plant species. PGs belong to one of the largest hydrolase families and have multiple functions in seed germination, organ abscission, pod and anther dehiscence, and xylem cell formation (Kramer and Redenbaugh 1994; Sitrit et al. 1996, 1999; Zhang et al. 2008; Huang et al. 2009a, b; Ogawa et al. 2009; Babu et al. 2013; Xiao et al. 2014). PGs are classified into endo-PGs, which hydrolyse the homogalacturonan polymers at random sites, and exo-PGs, which hydrolyse the free end of de-methylesterified homogalacturonan polymers (Hadfield and Bennett 1998; Markovic and Janecek 2001). Plant PGs have four conserved domains that are important for PG activity (Torki et al. 2000).

The full-length SP1 was identified as a homologue of plant exo-PG. Multiple sequence alignment analysis with other plant PGs suggested that the full-length SP1 has the four highly conserved domains (Fig. 3).

To determine whether buckwheat has other PGs similar to SP1, we searched the BGDB and found 51 putative PGs (Supplementary Table S4). The predicted full-length amino acid sequence of SP1 showed highest similarity to Fes_sc0006922.1.g000006.aua.1 (99%) and Fes_sc0001894.1.g000002.aua.1 (79%). Therefore, we considered SP1 to be identical to Fes_sc0006922.1.g000006.aua.1 and designated it *FePG1*.

Phylogenetic analysis with the 51 buckwheat PGs and 56 PGs from various plant species placed FePG1 in the same clade as BcMF2 and BcMF6 (Supplementary Table S3), which are orthologues of exo-PG in Chinese cabbage (*Brassica rapa*) and function in pollen development and floral morphology, *e.g*., style length and anther height (Huang et al. 2009a, b). Park et al. (2010) classified plant PGs into six clades (A to F). Clades A, B, and E encode endo-PGs, and Clades C and D encode exo-PGs. In our analysis, Clades C and D were combined into the same cluster, C, as in Huang et al. (2009a).

### Expression analysis of *FePG1*

**Fig. 4:**
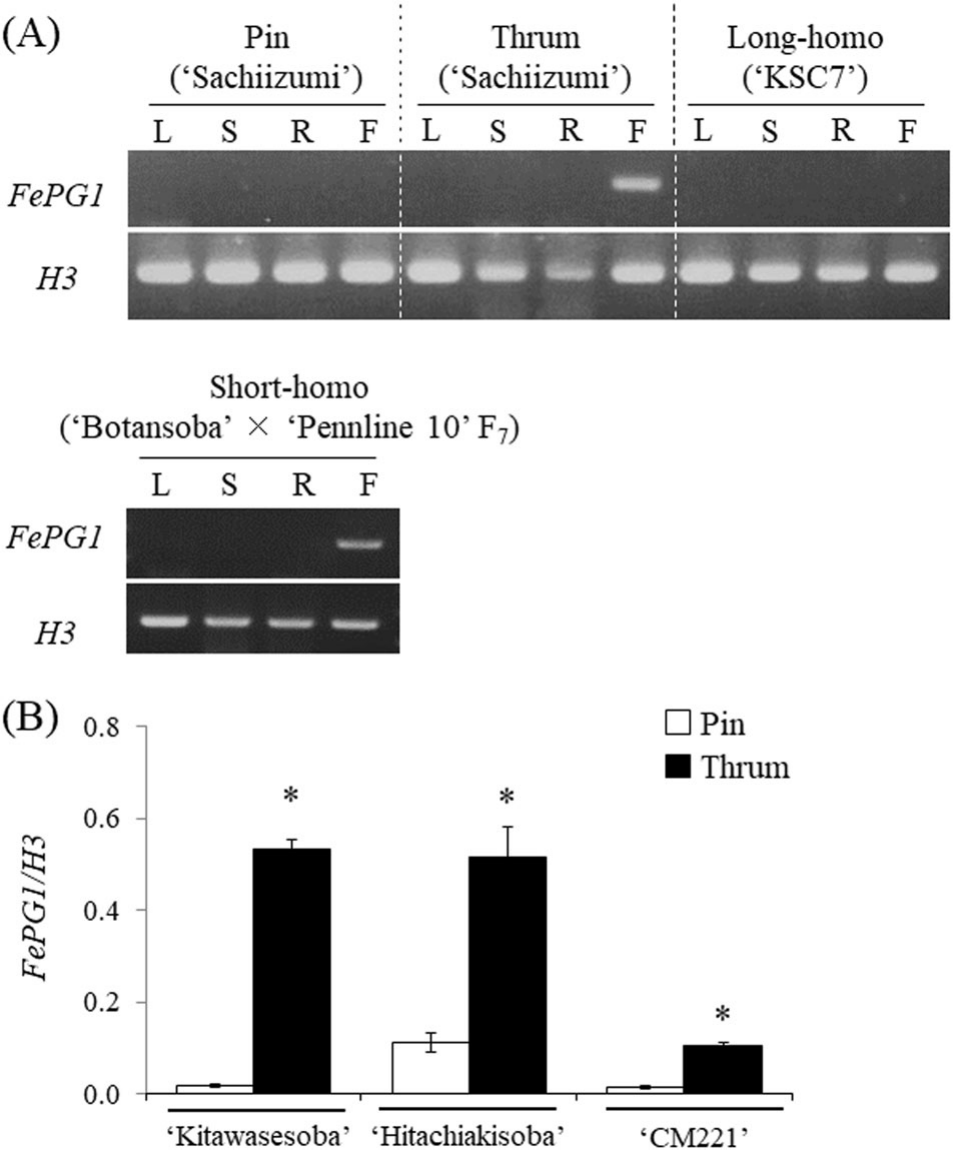
Relative expression levels of *FePG1*. **a** The mRNA abundances of *FePG1* in different organs of four floral morphotypes were detected by semi-qRT-PCR (40 cycles). The housekeeping gene *Histone H3* (*H3*) was used as a loading control. L, leaves; S, stems; R, roots; F, flowers. **b** The mRNA abundances of *FePG1* in pin and thrum styles were compared by real-time qRT-PCR. Values are relative to the *H3* transcript level. Data are means ± s.e. for three technical replicates (bulked RNA from at least four independent plants). *P < 0.05 (Student’s *t* test)

Many clade C exo-PGs are expressed mainly in pollen or the floral organs (Torki et al. 2000). Using semi-qRT-PCR, we determined the expression of *FePG1* in different organs (Fig. 4a). In the long-style plants (*i.e*., pin and long-homostyle), we detected no *FePG1* mRNA in any organ. In the short-style plants (*i.e*., thrum and short-homostyle), *FePG1* mRNA was specifically detected in flowers but not in vegetative organs. These results suggest that *FePG1* is expressed mainly in the floral organs, similar to other clade C *PG*s.

In the flowers of short-homostyle plants, which have short styles, as in thrum flowers, but low anthers, as in pin flowers, *FePG1* was expressed as in thrum plants (Fig. 4a). We compared the expression levels of *FePG1* in pin and thrum styles by using real-time qRT-PCR (Fig. 4b). In all three cultivars tested, the expression levels of *FePG1* in the styles were significantly higher in thrum plants than in pin plants. These results suggest that the expression level of *FePG1* is related to the floral morphotype, especially style length.

### Linkage analysis using an SC line

**Fig. 5:**
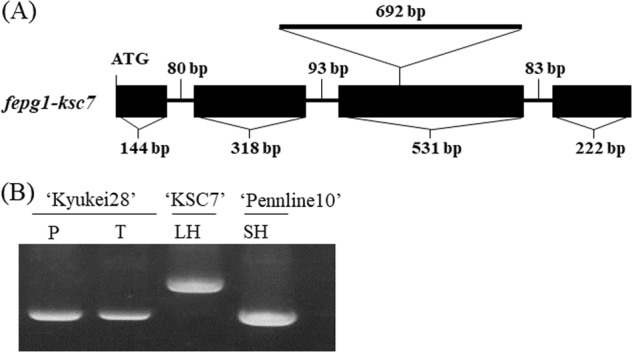
DNA polymorphism of the *FePG1* genomic region in ‘KSC7’ (long homostyle). **a** Gene structure of *FePG1* of ‘KSC7’. ‘KSC7’ has a 692-bp insertion in the 3rd exon. Black boxes, exons; bars, introns. (**b**) DNA polymorphism at the insertion region detected by agarose gel electrophoresis. P pin, T thrum, LH long homostyle, SH short homostyle

Sequence analysis revealed that ‘KSC7’ has a 692-bp insertion in the 3rd exon of *FePG1* (Fig. 5a). Using this sequence difference, we developed a marker to detect the *fepg1-ksc7* allele (Fig. 5b) and performed linkage analysis using two F_2_ populations to elucidate whether *FePG1* segregates with the *S* locus (Table 1). In both F_2_ populations, there were pin plants with an *fepg1-ksc7*/*fepg1-ksc7* genotype and long-homostyle plants with an *FePG1*/*FePG1* genotype. This result indicates that the *FePG1* locus is not linked to the *S* supergene.

**Table 1:** Linkage analysis between genotype of *FePG1* and floral morphs in F_2_ plants

Lines	Long homostyle^*a*^			Pin^a^			*χ* ^2^		
	AA	AB	BB	AA	AB	BB	Distylous^b^	*FePG1* ^c^	*χ* ^2L^
‘Kyukei 28’ × ‘KSC7’ (*n* = 130)	23	40	34	11	17	5	0.003 (0.95 < *P* < 0.98)	2.354 (0.30 < *P* < 0.40)	2.985 (0.20 < *P* < 0.30)
‘Kyushu 7’ × ‘KSC7’ (*n* = 97)	18	42	14	2	13	8	0.086 (0.70 < *P* < 0.80)	1.825 (0.40 < *P* < 0.50)	2.814 (0.20 < *P* < 0.30)

^a^AA, *fepg1-ksc7*/*fepg1-ksc7* genotype; AB, *FePG1*/*fepg1-ksc7* genotype; BB, *FePG1*/*FePG1* genotype

^b^Expected segregation rate of floral morphs long homostyle: pin = 3:1

^c^Expected segregation rate of *FePG1* genotypes AA: AB: BB = 1:2:1

### Search for *FePG1* promoter

**Fig. 6:**
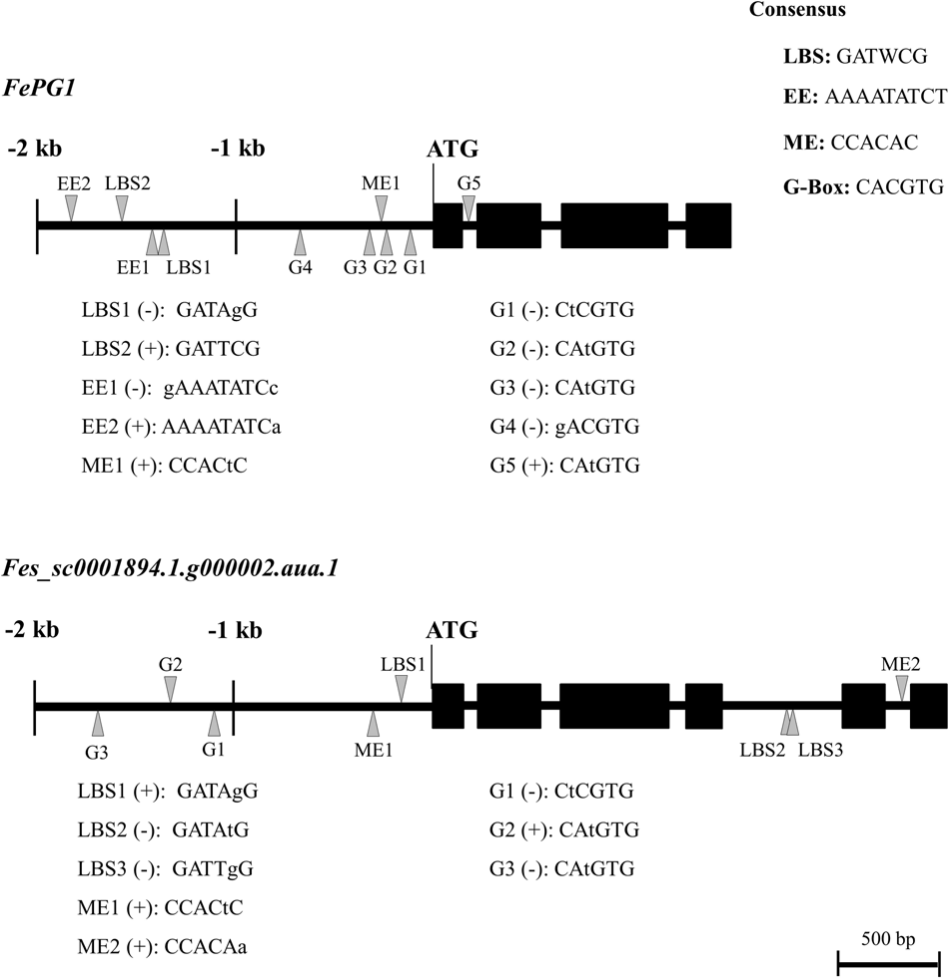
*Cis*-elements of *FePG1* and *Fes_sc0001894.1.g000002.aua.1* A 2-kb region upstream of the translation initiation site and intron regions were analyzed. Triangles indicate *cis*-elements: LUX-binding site (LBS), evening element (EE), morning element (ME), and G-box (G). The lowercase letters are sequences different from the consensus sequence of each *cis*-element; + , forward strand; –, reverse strand

Although the above linkage analysis revealed that *FePG1* is not a member of the *S* supergene, the levels of protein and mRNA of *FePG1* were certainly associated with floral morphology (Figs. 2 and 4). These results suggest that *FePG1* could be regulated by the *S* supergene to play a role in determining style length. Therefore, we analyzed the promoter sequence to reveal whether *FePG1* has *cis*-elements that can be regulated by the *S* supergene. One candidate gene in the *S* supergene in common buckwheat is *S-ELF3*, a homologue of *Arabidopsis ELF3* (Yasui et al. 2012). In *Arabidopsis*, ELF3 is a core circadian clock component and functions in flowering repression (Zagotta et al. 1996; Liu et al. 2001), diurnal control of hypocotyl growth (Nusinow et al. 2011), thermo-responsive hypocotyl growth (Box et al. 2015; Raschke et al. 2015), and salt tolerance (Sakuraba et al. 2017). *Arabidopsis* ELF3 interacts with ELF4 and LUX ARRHYTHMO (LUX), and the ELF4–ELF3–LUX complex binds to the promoters of circadian clock genes such as *PHYTOCHROME-INTERACTING FACTOR 4* (*PIF4*), *PIF5*, and *PSEUDO-RESPONSE REGULATOR 9* to regulate their expression in a circadian manner (Helfer et al. 2011; Herrero et al. 2012). The conserved target sequences of the ELF4–ELF3–LUX complex are the LUX-binding site (LBS; GATWCG), evening element (AAAATATCT), morning element (CCACAC), and G-box (CACGTG) (Harmer et al. 2000; Quail 2000; Michael et al. 2008; Helfer et al. 2011; Herrero et al. 2012; Huang et al. 2012). We searched for these four *cis*-elements in the 2-kb region upstream from the translation initiation site and in intron regions of *FePG1*. We detected one LBS (−1580 bp) and one putative LBS (−1390 bp), two putative evening elements (−1833 and −1422 bp), one putative morning element (−247 bp), and four putative G-box elements (−668, −320, −234, and −117 bp) (Fig. 6). We also searched the same promoter and intron regions of *Fes_sc0001894.1.g000002.aua.1*, which encodes a protein highly similar to FePG1 (Fig. 6), and found that it do not contain the evening element. These results support the hypothesis that the expression level of *FePG1* is specifically regulated by S-ELF3, which is a candidate gene of the *S* supergene.

## Discussion

### Possible role of a short-style–specific protein inferred from sequence similarity

Here we identified a short-style–specific protein in buckwheat by comparing protein profiles between the upper pistils of pin (long style) and thrum (short style) plants by 2D-PAGE. This protein was found to be a PG, and was designated FePG1 (Figs. 2 and 3). PGs are pectin hydrolytic enzymes involved in various developmental processes (Kramer and Redenbaugh 1994; Sitrit et al. 1996, 1999; Zhang et al. 2008; Huang et al. 2009a, b; Ogawa et al. 2009; Babu et al. 2013; Xiao et al. 2014). In the phylogenetic tree, FePG1 was placed in clade C (Supplementary Fig. S1), which includes exo-PGs that may be involved in pollen development and floral morphology, *e.g*., style length and anther height (Athanasiou et al. 2003; Zhang et al. 2008; Huang et al. 2009a, b; Carvajal et al. 2014; Yu et al. 2014). Therefore, FePG1 may have a role in the development of floral morphology or be related to self-incompatibility through pollen tube development.

We detected a 15-kDa truncated form but not full-length FePG1 by 2D-PAGE. Because transcripts encoding the full-length FePG1 protein were detected in short-style plants, our inability to detect full-length FePG1 might be due to the presence of many other protein spots at its expected position.

### Role of PG in other plant species and comparison with FePG1

Huang et al. (2009a) developed RNAi transgenic plants of *BcMF2*, which is an exo-PG orthologue in Chinese cabbage. These plants have shorter filaments, shrunken anthers, and less pollen than control plants, so this abnormal pollen development may lower fruit set in the transgenic plants. In Chinese cabbage (Huang et al. 2009a), knockdown of the exo-PG caused shorter filaments, whereas in common buckwheat, FePG1 specifically accumulated in the styles of thrum flowers, and *FePG1* was expressed in the styles of short-homostyle and thrum plants (Fig. 4). Thus, functional *FePG1* may shorten the length of the style. Wang et al. (2016) identified several PGs in soybean (G*lycine max*) and reported that the PG031 gene (*GmPG031*) has different alleles, *GmPG031*^*289H*^ and *GmPG031*^*289Y*^, both of which show flower-specific expression patterns. Constitutive expression of *GmPG031*^*289H*^ in transgenic *Arabidopsis* plants significantly increased the silique length relative to the wild type, whereas constitutive expression of *GmPG031*^*289Y*^ significantly decreased it (Wang et al., 2016). Considering that these variants differ by only one amino acid (H or Y) caused by a SNP, the phenotype caused by PGs may depend on subtle changes in the coding sequence.

### Effect of *FePG1* expression on the style length of pin plants

The low levels of *FePG1* mRNA we detected in the bulked styles of pin plants of ‘HTC’ (Fig. 4b) may be caused by an increase in the proportion of abnormal flowers with short styles because the experiment was performed in summer, whereas ‘HTC’ is a medium-autumn ecotype. Buckwheat has four ecotypes (summer, medium-summer, medium-autumn, and autumn) with different sensitivities to daylength; cultivation of an autumn-type buckwheat in summer increases the proportion of abnormal flowers (Nagatomo 1961, Lachmann and Adachi 1990, Guan and Adachi 1992). Although we did not measure the style length of ‘HTC’, the samples could include such abnormal flowers.

Tatebe (1953) found a SC common buckwheat plant with pin flowers and obtained self-fertilised seeds. All progeny of this plant had pin flowers, but their styles were shorter than those of other pin plants, and were self-compatible. Matsui et al. (2004) reported that ‘Pennline 10’, which is a short-homostyled SC common buckwheat, should have the *ss* genotype, the same as pin. They suggested that this self-compatibility and style length are regulated by novel modifier genes that are outside of the *S* supergene. We detected abundant *FePG1* mRNA in another short-homostyled SC line (Fig. 4a). We hypothesise that pin plants can develop short styles when they express abundant *FePG1* mRNA, and the resulting short-styled pin plants acquire self-compatibility. More detailed experiments on short-styled pin plants are needed to reveal the relationship between the expression of *FePG1*, style length, and self-compatibility.

### *FePG1* is not an *S* locus gene but could function downstream of this locus

Linkage analysis revealed that *FePG1* was not a member of the *S* supergene (Table 1); however, the levels of *FePG1* mRNA and protein were certainly associated with the floral morphology (Figs. 2 and 4). These results suggest that *FePG1* could have a role in the heteromorphic SI system and be regulated by the *S* supergene. The promoter analysis revealed that *FePG1* has ten putative binding sites for the protein encoded by *S-ELF3*, which is a candidate for a gene in the *S* supergene in common buckwheat (Fig. 6). Like *FePG1*, *S-ELF3* is specifically expressed in the floral organs (pistil) of short-style plants and not in the vegetative organs (Fig. 4; Yasui et al. 2012). These similar expression profiles suggest that the expression levels of *FePG1* might be upregulated by *S-ELF3*.

In *Turnera*, two PG homologues, TsPG (a style-specific PG) and TsPP (a pollen-specific PG), have been identified as distyly-related proteins (Athanasiou and Shore 1997; Athanasiou et al. 2003; Khosravi et al. 2003; Tamari and Shore 2004). The gene encoding TsPG is located 4.6 cM distal to the *distyly* locus, and TsPG protein does not accumulate in long-styled plants, although these plants carry the allele (Athanasiou et al. 2003). Tamari and Shore (2006) detected two different alleles—*TsPG*^*S*^, derived from long-styled plants, and *TsPG*^*B*^, derived from short-styled plants—and measured accumulation of each TsPG protein in long- and short-styled plants derived from recombination between the *distyly* and *TsPG* loci. They found that long-styled plants possessing the allele of *TsPG*^*B*^ derived from a chromosome bearing the *S* allele did not accumulate TsPG^B^ protein; in contrast, short-styled plants possessing the allele of *TsPG*^*S*^ derived from a chromosome bearing the *s* allele accumulated TsPG^S^ protein. They postulated that the dominant *S* allele upregulates the expression of *TsPG*. Interestingly, TsPG also had a truncated N-terminal amino acid sequence of 15-kDa and ca. 35-kDa protein (Khosravi et al. 2003). These results are similar to our results for buckwheat, although we detected only a 15-kDa PG protein.

In *Primula*, the *S* locus has been identified by RNA sequencing and *de novo* sequence assembly (Nowak et al. 2015, Li et al. 2015, 2016; Huu et al. 2016). Five ORFs encoding a protein with a conserved cysteine motif (CCM^T^), a homolog to MADS-box transcription factors (GLO^T^), cytochrome P450 similar to *Arabidopsis* CYP72B1 (CYP^T^), a Pumilio-like RNA-binding protein (PUM^T^), and a protein similar to the *Arabidopsis* Kiss-Me-Deadly Kelch repeat F Box protein (KFB^T^) have been detected as putative elements of the *S* supergene (Li et al. 2016). Because these genes may regulate hormone pathways or transcription factors, many genes outside of the *S* locus would be expected to be under their control. Burrows and McCubbin (2018) performed RNA sequencing of floral buds of pin and thrum morphs in *P. vulgaris* at early and late stages to detect *S* locus–regulated genes; they identified 540 genes that were differentially expressed between pin and thrum in the early stage and 3101 in the late stage. Two PG orthologues (transcript numbers C189 and C7260) were expressed at lower levels in thrum than in pin in the early stage. In the late stage, two PG orthologues (C866 and C6779) were more highly expressed in thrum than in pin, and two PG orthologues (C3171 and C11125) were expressed at lower levels in thrum than in pin (Burrows and McCubbin 2018). Thus, some *P. vulgaris* PG genes are differentially expressed depending on flower shape.

Our results, together with the above data on *Turnera* and *Primula*, support the notion that *FePG1* is a heteromorphic self-incompatibility–related factor that influences the style length and/or pollen tube development, and that the expression of *FePG1* is regulated by the buckwheat *S* supergene. Future studies are needed to clarify the role of *FePG1* and whether *S-ELF3* or other genes in the *S* supergene can upregulate its expression.

### Data archiving

Data available from the Dryad Digital Repository: 10.5061/dryad.3gm4500.

## Supplementary information

Supplementary Fig. S1

Supplementary Table S1

Supplementary Table S2

Supplementary Table S3

Supplementary Table S4
