# Supplementary material for: Identification of a gene encoding polygalacturonase expressed specifically in short styles in distylous common buckwheat (Fagopyrum esculentum)
Source: Heredity (Edinb). 2019 May 10;123(4):492–502. doi: 10.1038/s41437-019-0227-x (PMC6781162; doi:10.1038/s41437-019-0227-x)
Supplement: Supplementary file 1 — Supplementary Fig. S1 [file 41437_2019_227_MOESM1_ESM.pdf]

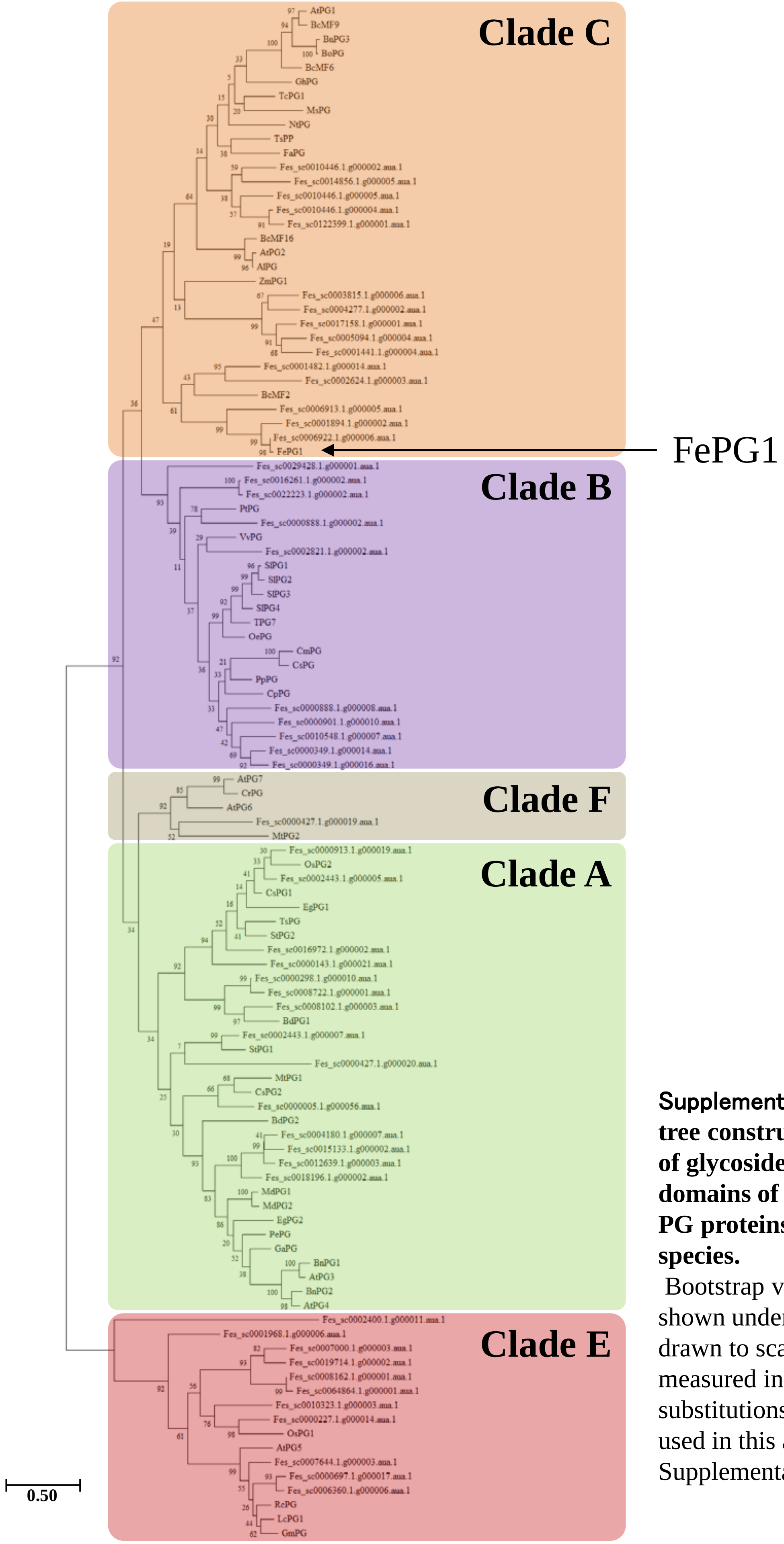

Supplementary Fig. S1. Phylogenetic tree constructed with the sequences of glycoside hydrolase family 28 domains of 51 buckwheat PG and 56 PG proteins from various plant species.

Bootstrap values (1000 replicates) are shown under the branches. The tree is drawn to scale, with branch lengths measured in the number of substitutions per site. The PG proteins used in this analysis are listed in Supplementary Tables S3 and S4
