# Supplementary material for: Identification of a gene encoding polygalacturonase expressed specifically in short styles in distylous common buckwheat (Fagopyrum esculentum)
Source: Heredity (Edinb). 2019 May 10;123(4):492–502. doi: 10.1038/s41437-019-0227-x (PMC6781162; doi:10.1038/s41437-019-0227-x)
Supplement: Supplementary file 2 — Supplementary Table S1 [file 41437_2019_227_MOESM2_ESM.pdf]

Supplementary Table S1. Plant materials used in proteomic analysis, quantitative RT-PCR, and linkage analysis.

Lines	Floral morphology	Discription
**For proteomic analysis**		
Botansoba (BTN)	Heterostyly	Japanese cultivar
Hashikamiwase	Heterostyly	Japanese cultivar
Hitachiakisoba (HTC)	Heterostyly	Japanese cultivar
Shinano 1	Heterostyly	Japanese cultivar
Asahimura-zairai 3	Heterostyly	Japanese landrace
Kanoya-zairai	Heterostyly	Japanese landrace
Kugino-zairai Norin-	Heterostyly	Japanese landrace
PL1	Long-homostyle	Matsui *et al* . (2008)
**For quantitative RT-PCR**		
Sachiizumi	Heterostyly	Japanese cultivar
Kyukei SC7 (KSC7)	Long-homostyle	Developed from "Norin-PL1"
L21SH	Short-homostyle	F_7 of Botansoba × Pennline 10 (SC, Short-homostyle line developed by Marshall *et al.* , 1970)
Kitawasesoba	Heterostyly	Japanese cultivar
Hitachiakisoba	Heterostyly	Japanese cultivar
CM 221	Heterostyly	Chinese cultivar
**For linkage analysis**		
Kyukei 28(Pin)/KSC7(LH)	Segregate Pin and Long-homostyle	F_2 of Kyukei 28 × Kyukei SC7
Kyusyu 7(Pin)/KSC7(LH)	Segregate Pin and Long-homostyle	F_2 of Kyusyu 7 × Kyukei SC7
