# Supplementary material for: Identification of a gene encoding polygalacturonase expressed specifically in short styles in distylous common buckwheat (Fagopyrum esculentum)
Source: Heredity (Edinb). 2019 May 10;123(4):492–502. doi: 10.1038/s41437-019-0227-x (PMC6781162; doi:10.1038/s41437-019-0227-x)
Supplement: Supplementary file 3 — Supplementary Table S2 [file 41437_2019_227_MOESM3_ESM.pdf]

Supplementary Table S2. Sequences of primers used for cloning, quantitative RT-PCR, and linkage analysis.

Objectives	Forward primer	Forward primer sequence (5'–3')	Reverse primer	Reverse primer sequence (5'–3')	Description
Cloning of SP1	dgSIspAN1 (APDERLFN)	GCNCCNGAYGARMGNYTNTTYAA	dgspAMR1 (EACNYVGK)	YTTNCCNACRTARTTRCANGCYTC	Degenerate PCR for cloning of SP1
	dgSIspAN2 (DERLFNVV)	GAYGARMGNYTNTTYAAYGTNGT	dgspAMR3 (AWGEACNY)	RTARTTRCANGCYTCNCCCCANGC	Degenerate PCR for cloning of SP1
	SPAF005	GGTGACAAAGCTAATAATGTAG			3' RACE
	SPAR001b	TCACCCCAAGCTCTAGTGAAAGC			Genome walking to identify the 5' region
	SPAR002	GCTTTTACACGGACCCGAAAAC			Genome walking to identify the 5' region
Gene expression analysis	sRT_F1	GACTATGGTGCTAAAACCGGTGA	sRT_R1	GCTTTTACACGGACCCGAAAAC	*FePG1* semi-qRT-PCR
	qRT_F1	CCGGGTTTTTGTTCAAGGACATCG	qRT_R1	GCCGAACTGCACACCAAGC	*FePG1* real-time qRT-PCR
	H3F	GAAATTCGCAAGTACCAGAAGAG	H3R	CCAACAAGGTATGCCTCAGC	Histone H3 (reference)
Linkage analysis	LK_F1	ATGGCCACGTTTTGGATTTGTATTCTTGC	LK_R1	TTAGCAGTTAACAGGAGGAAGCTGG	*FePG1* _KSC7
	LK_F2	CACCATCGATGGTCAAGGCAACAA	LK_R2	TGGTCTAGGGTTCAAATCCC	*FePG1* _KSC7_insert_1
	LK_F3	ACGAACGTGCTAGTCCGAAGCA	LK_R3	ATGGGGGTGGCTTTTCCGTTG	*FePG1* _KSC7_insert_2
	LK_F4	GACTATGGTGCTAAAACCGGTGA	LK_R4	GCCTCAGGCCAACATATCGTAG	*FePG1* _KSC7_detection

PCR products amplified with the set of degenerate primers, dgSIspAN1 (APDERLFN) and dgspAMR1 (EACNYVGK), were diluted 100-fold and used for degenerate PCR. Electrophoretic analysis of DNA fragments amplified from genomic DNA and cDNA by degenerate PCR with the primer set of dgSIspAN2 (DERLFNVV) and dgspAMR2 (AWGEACNY) showed a ca. 200-bp band and a ca. 100-bp band, respectively. After determining the nucleotide sequence of the region between the primers dgSIspAN2 and dgspAMR2, we performed 3′ RACE with the primer SPAF005, which was designed in the region between gSIspAN2 and dgspAMR2, and the primers of 3′- Full RACE Core Set (Takara, Ostu, Japan). The sequence of the 5′ region was then determined by genome walking with the Gene Racer Kit using two different primers.
