# Supplementary material for: Identification of a gene encoding polygalacturonase expressed specifically in short styles in distylous common buckwheat (Fagopyrum esculentum)
Source: Heredity (Edinb). 2019 May 10;123(4):492–502. doi: 10.1038/s41437-019-0227-x (PMC6781162; doi:10.1038/s41437-019-0227-x)
Supplement: Supplementary file 4 — Supplementary Table S3 [file 41437_2019_227_MOESM4_ESM.pdf]

Supplementary Table S3. Accession numbers for amino acid sequences used for phlogenetic analysis

Name	Species	Accession number	Endo / Exo	Clade in Fig. 4
AtPG2	*Arabidopsis thaliana*	CAA51692.1	Exo	**C**
AlPG	*Arabidopsis lyrata*	EFH61264.1		**C**
BcMF16	*Brassica campestris*	ADJ68232.1	Exo	**C**
TsPP	*Turnera subulata*	AAO24261		**C**
GhPG	*Gossypium hirsutum*	AAA82167.1		**C**
NtPG	*Nicotiana tabacum*	Q05967.1		**C**
TcPG1	*Theobroma cacao*	EOX93416.1		**C**
MsPG	*Medicago sativa*	CAA72003.1		**C**
FaPG	*Fragaria ananassa*	ABE77145.1		**C**
ZmPG1	*Zea mays*	P26216.1	Exo	**C**
BcMF6	*Brassica campestris*	ACP74159.1		**C**
AtPG1	*Arabidopsis thaliana*	NP_187439.1		**C**
BcMF9	*Brassica campestris*	ABN13878.1		**C**
BnPG3	*Brassica napus*	CDY22140.1		**C**
BoPG	*Brassica oleracea*	AAM74219.1		**C**
BcMF2	*Brassica campestris*	ABW24665.1		**C**
FePG1	*Fagopyrum esculentum*	LC431856		**C**
CmPG	*Cucumis melo*	AAC26511.1		**B**
CsPG	*Cucumis sativus*	XP_004133718.1		**B**
PtPG	*Populus trichocarpa*	XP_006372694.1		**B**
VvPG	*Vitis vinifera*	XP_010646462.1		**B**
PpPG	*Prunus persica*	CAA54150.1	Endo	**B**
CpPG	*Carica papaya*	ACH82233.1		**B**
OePG	*Olea europaea*	ACA49228.1		**B**
TPG7	*Solanum lycopersicum*	NP_001307785		**B**
SlPG4	*Solanum lycopersicum*	AAB09576.1		**B**
SlPG3	*Solanum lycopersicum*	AAC28902.2		**B**
SlPG1	*Solanum lycopersicum*	AAC28903.1		**B**
SlPG2	*Solanum lycopersicum*	AAC28904.1		**B**
OsPG2	*Oryza sativa*	AAT39158.1		**A**
CsPG1	*Citrus sinensis*	XP_006483884.1		**A**
TsPG	*Turnera subulata*	AAO24262		**A**
StPG2	*Solanum tuberosum*	XP_006359737.1		**A**
EgPG1	*Eucalyptus grandis*	XP_010041884.1		**A**
BdPG1	*Brachypodium distachyon*	XP_010231630.1		**A**
MtPG1	*Medicago truncatula*	KEH39601.1		**A**
CsPG2	*Citrus sinensis*	ABM67700.1		**A**
StPG1	*Solanum tuberosum*	XP_006350275.1		**A**
BdPG2	*Brachypodium distachyon*	XP_003558488.1		**A**
BnPG1	*Brassica napus*	CAA65072.1		**A**
AtPG3	*Arabidopsis thaliana*	NP_191310.1		**A**
BnPG2	*Brassica napus*	CAC05658.1	Endo	**A**
AtPG4	*Arabidopsis thaliana*	NP_850359.1	Endo	**A**
EgPG2	*Eucalyptus grandis*	XP_010032438.1		**A**
MdPG1	*Malus domestica*	AAA74452.1	Endo	**A**
MdPG2	*Malus domestica*	XP_008356211.1		**A**
GaPG	*Gossypium arboreum*	KHG11893.1		**A**
PePG	*Populus euphratica*	XP_011048121.1		**A**
MtPG2	*Medicago truncatula*	AES59077.2		**F**
AtPG6	*Arabidopsis thaliana*	CAA20037.1		**F**
AtPG7	*Arabidopsis thaliana*	NM_123851		**F**
CrPG	*Capsella rubella*	XP_006279827.1		**F**
OsPG1	*Oryza sativa*	AAT44156.1		**E**
AtPG5	*Arabidopsis thaliana*	ABE65854.1		**E**
GmPG	*Glycine max*	XP_003535598.1		**E**
LcPG	*Litchi chinensis*	AFW04075.1		**E**
RcPG	*Ricinus communis*	XP_002513653.1		**E**
