# Supplementary material for: Identification of a gene encoding polygalacturonase expressed specifically in short styles in distylous common buckwheat (Fagopyrum esculentum)
Source: Heredity (Edinb). 2019 May 10;123(4):492–502. doi: 10.1038/s41437-019-0227-x (PMC6781162; doi:10.1038/s41437-019-0227-x)
Supplement: Supplementary file 5 — Supplementary Table S4 [file 41437_2019_227_MOESM5_ESM.pdf]

Supplementary Table S4. Buckwheat PGs used for phylogenetic analysis.

ID	Length (aa)	Clade in Fig. S1
Fes_sc0010446.1.g000002.aua.1	108	**C**
Fes_sc0014856.1.g000005.aua.1	267	**C**
Fes_sc0010446.1.g000005.aua.1	135	**C**
Fes_sc0010446.1.g000004.aua.1	317	**C**
Fes_sc0122399.1.g000001.aua.1	125	**C**
Fes_sc0003815.1.g000006.aua.1	965	**C**
Fes_sc0004277.1.g000002.aua.1	508	**C**
Fes_sc0017158.1.g000001.aua.1	866	**C**
Fes_sc0005094.1.g000004.aua.1	395	**C**
Fes_sc0001441.1.g000004.aua.1	395	**C**
Fes_sc0001482.1.g000014.aua.1	515	**C**
Fes_sc0002624.1.g000003.aua.1	538	**C**
Fes_sc0006913.1.g000005.aua.1	415	**C**
Fes_sc0001894.1.g000002.aua.1	525	**C**
Fes_sc0006922.1.g000006.aua.1 (FePG1)	404	**C**
Fes_sc0029428.1.g000001.aua.1	403	**B**
Fes_sc0016261.1.g000002.aua.1	429	**B**
Fes_sc0022223.1.g000002.aua.1	105	**B**
Fes_sc0000888.1.g000002.aua.1	393	**B**
Fes_sc0002821.1.g000002.aua.1	445	**B**
Fes_sc0000888.1.g000008.aua.1	402	**B**
Fes_sc0000901.1.g000010.aua.1	390	**B**
Fes_sc0010548.1.g000007.aua.1	244	**B**
Fes_sc0000349.1.g000014.aua.1	394	**B**
Fes_sc0000349.1.g000016.aua.1	393	**B**
Fes_sc0000427.1.g000019.aua.1	313	**F**
Fes_sc0000913.1.g000019.aua.1	519	**A**
Fes_sc0002443.1.g000005.aua.1	522	**A**
Fes_sc0016972.1.g000002.aua.1	603	**A**
Fes_sc0000143.1.g000021.aua.1	474	A
Fes_sc0000298.1.g000010.aua.1	474	**A**
Fes_sc0008722.1.g000001.aua.1	76	**A**
Fes_sc0008102.1.g000003.aua.1	480	**A**
Fes_sc0002443.1.g000007.aua.1	452	**A**
Fes_sc0000427.1.g000020.aua.1	94	**A**
Fes_sc0000005.1.g000056.aua.1	452	**A**
Fes_sc0004180.1.g000007.aua.1	127	**A**
Fes_sc0015133.1.g000002.aua.1	440	**A**
Fes_sc0012639.1.g000003.aua.1	190	**A**
Fes_sc0018196.1.g000002.aua.1	419	**A**
Fes_sc0002400.1.g000011.aua.1	474	**E**
Fes_sc0001968.1.g000006.aua.1	483	**E**
Fes_sc0007000.1.g000003.aua.1	442	**E**
Fes_sc0019714.1.g000002.aua.1	276	**E**
Fes_sc0008162.1.g000001.aua.1	403	**E**
Fes_sc0064864.1.g000001.aua.1	112	**E**
Fes_sc0010323.1.g000003.aua.1	540	**E**
Fes_sc0000227.1.g000014.aua.1	479	**E**
Fes_sc0007644.1.g000003.aua.1	480	**E**
Fes_sc0000697.1.g000017.aua.1	476	**E**
Fes_sc0006360.1.g000006.aua.1	501	**E**
